# Systemic Emergencies in COVID-19 Patient: A Pictorial Review

**DOI:** 10.3390/tomography8020084

**Published:** 2022-04-06

**Authors:** Marco Albanesi, Diletta Cozzi, Edoardo Cavigli, Chiara Moroni, Gianluca Frezzetti, Lina Bartolini, Vittorio Miele

**Affiliations:** 1 Department of Emergency Radiology, University Hospital Careggi, L.go Brambilla 3, 50134 Florence, Italy; albanesima@aou-careggi.toscana.it (M.A.); caviglie@aou-careggi.toscana.it (E.C.); moronic@aou-careggi.toscana.it (C.M.); frezzettig@aou-careggi.toscana.it (G.F.); bartolinili@aou-careggi.toscana.it (L.B.); mielev@aou-careggi.toscana.it (V.M.); 2 SIRM Foundation, Via della Signora, 20019 Milan, Italy

**Keywords:** infection, emergencies, computed tomography, ultrasound, embolism, stroke

## Abstract

Since the first report of the outbreak in Wuhan, China in December 2019, as of 1 September 2021, the World Health Organization has confirmed more than 239 million cases of the novel coronavirus (SARS-CoV-2) infectious disease named coronavirus disease 2019 (COVID-19), with more than 4.5 million deaths. Although SARS-CoV-2 mainly involves the respiratory tract, it is considered to be a systemic disease. Imaging plays a pivotal role in the diagnosis of all manifestations of COVID-19 disease, as well as its related complications. The figure of the radiologist is fundamental in the management and treatment of the patient. The authors try to provide a systematic approach based on an imaging review of major multi-organ manifestations of this infection.

## 1. Introduction

In December 2019, a new virus, SARS-CoV-2 (Severe Acute Respiratory Syndrome Coronavirus 2), started from China and had a rapid global diffusion [1]. This new coronavirus was identified to be a highly infectious disease, commonly called Coronavirus Disease 2019 (COVID-19), and, due to a rapid worldwide spread, the World Health Organization (WHO) declared a global pandemic on 11 March 2020 [2]. The exponential increase in the number of COVID-19 patients soon saturated emergency rooms and, subsequently, due to the wide spectrum of complications, hospitalization was necessary in the ordinary ward or intensive care. It was therefore necessary to reconvert hospital facilities, including radiology departments, to contain intra-hospital infection and protect health professionals [3,4]. SARS-CoV-2 was primarily considered a viral respiratory disease; however, scientific evidence has shown that SARS-CoV-2 causes multi-organ dysfunction [5,6,7]. The cornerstone of COVID-19 diagnosis is RT-PCR from the oral-nasopharyngeal swab. RT-PCR has very high specificity but limited sensitivity (61–70%); computed tomography (CT) outperforms RT-PCR sensitivity even in the early stages of the COVID-19 disease [8,9,10]. The early identification of pneumonia and multi-organ complications can modify the course of the disease and therefore improve the patient’s prognostic outcome. Certainly, in this challenge, diagnostic imaging and the radiologist have a pivotal role [11]. In the first months of the pandemic, the therapeutic management of COVID-19 and its sequelae was limited to supportive and experimental therapies. After months of research and collaboration between several nations, vaccines have been developed and have allowed a reduction in the incidence of infection and a lower occurrence of complications in previously vaccinated infected subjects [12,13].

The purpose of this essay is to describe the main emergencies related to COVID-19 infection and their radiological signs, primarily based on CT exams.

## 2. COVID-19 and Lung Disease

### 2.1. Pneumonia

**Figure 1:**
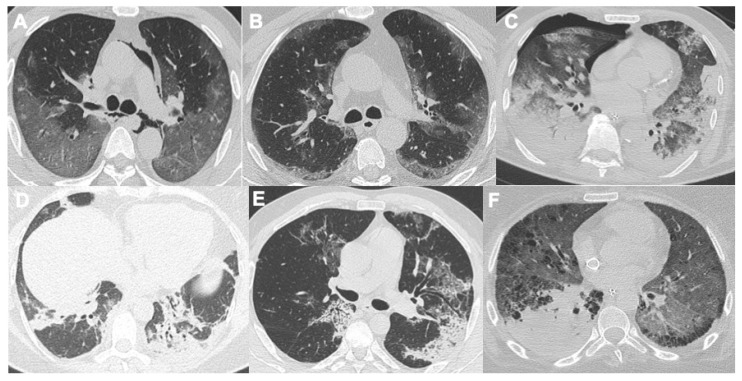
Six cases of interstitial pneumonia related to COVID-19 and their evolution. (**A**,**B**) typical features of the acute phase, with peripheral ground-glass opacities; in (**A**), it is also possible to recognize pneumomediastinum. (**C**) shows the evolution of the diffuse alveolar damage, usually after 7–10 days the infection, evolving through acute distress respiratory syndrome (ARDS); right pneumothorax due to barotrauma is also present. (**D**,**E**) show the late phase, with a typical “organizing pneumonia” pattern; (**F**) is a case of bacterial superinfection in the right lower lobe in a patient with venovenous ECMO and nasogastric tube.

Respiratory involvement is the most commonly associated manifestation of SARS-CoV-2. It is characterized by fever, cough, shortness of breath, fatigue, chest pain, and dyspnea. Pneumonia is the most serious symptom. Chest X-ray (CXR) and CT are essential to confirm the diagnosis of SARS-CoV-2 pneumonia. Radiographic features include consolidation, ground-glass opacities (GGO), pulmonary nodules, and reticular–nodular opacities. Moreover, on CXRs, there is a specific distribution of findings: the peripheral and lower zones are most commonly involved. Bilateral involvement is more frequent than unilateral [14,15,16,17,18]. CT, with a sensitivity between 69 and 98%, is the gold standard among imaging methods in identifying thoracic and lung parenchyma involvement [9,19]. The most frequent findings are ground-glass opacities, and consolidation and thickening of the bronchial walls. Less frequently, ground-glass opacities are associated with thickening of the interlobular septa (crazy-paving pattern), a reverse halo sign, lymphadenopathy, and pleural effusion [20,21] (Figure 1). In some cases, there might be a bacterial superinfection (Figure 1F), with classical alveolar and bronchial parenchymal involvement and in some cases lobar pneumonia: this complication has to be treated promptly with specific antibiotics in order to avoid the evolution into acute respiratory distress syndrome (ARDS).

Some studies have focused on the prognostic role of CT and evaluated lung parenchyma involvement, with visual scores and using artificial intelligence (AI) technology, generally with good correlations with patient outcome [22,23,24,25]. Certainly, AI with its quantification of pulmonary involvement and can be helpful in risk assessment. Pulmonary ultrasound, despite being a relatively recent application, plays a central role in the diagnosis and follow-up of various diseases, especially in critically ill patients [26]. During the course of the pathology, it is possible to identify the first phase with focal areas of fixed B lines, a phase of numerical increase of the B lines up to the “white lung” with small sub-pleural thickenings up to the evidence of real posterior consolidations [27]. A lack of use of ionizing radiation is fundamental in the most vulnerable patients, such as pregnant women, in whom lung ultrasound is a safe tool for monitoring the evolution of COVID-19 disease [28]. As underlined by Di Serafino et al., a lung ultrasound is not an anatomical ultrasound but a clinical interpretation of some artefacts; moreover, it is not an alternative imaging exam to a chest X-ray or CT but should be strongly integrated with them [29].

### 2.2. Acute Respiratory Disease Syndrome

**Figure 2:**
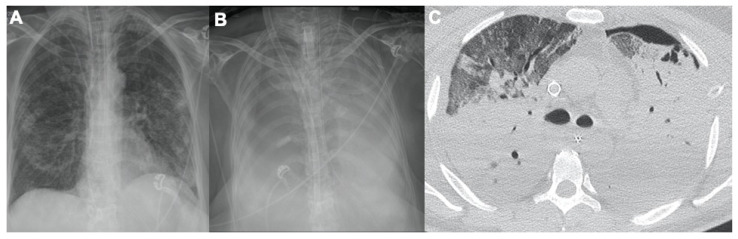
Evolution of COVID-19 pneumonia in the same patient (52-year-old male). (**A**) Day 9 from infection: peripheral opacities, 2 central venous catheters, endotracheal tube and nasogastric tube. (**B**) Day 14 from infection: both lungs are fully consolidated with bilateral pleural effusion, V-V ECMO, and tracheostomy. (**C**) CT at day 20, ARDS with left pneumothorax and chest tube positioned.

One of the most common respiratory complications of COVID-19 infection is ARDS: this is a clinical diagnosis classified on the degree of hypoxia. Imaging plays a supporting role in its diagnosis and management [27,30]. Usually, a week after onset of the disease, ARDS could develop in 17% of patients: 65% of these become worse and die due to multiorgan dysfunction [31]. Chest X-rays and CT scans can help with the identification of additional underlying causes of symptoms of ARDS, such as bacterial superinfection or congestive heart failure. In the acute phase of ARDS, chest X-ray may show bilateral airspace opacities with air bronchograms. In the acute exudative phase of ARDS, CT demonstrates diffuse GGOs with a posterior and basal predominance; a crazy-paving pattern may also be depicted [20,32] (Figure 2). Moreover, patchy and bilateral consolidations may be present.

**Figure 3:**
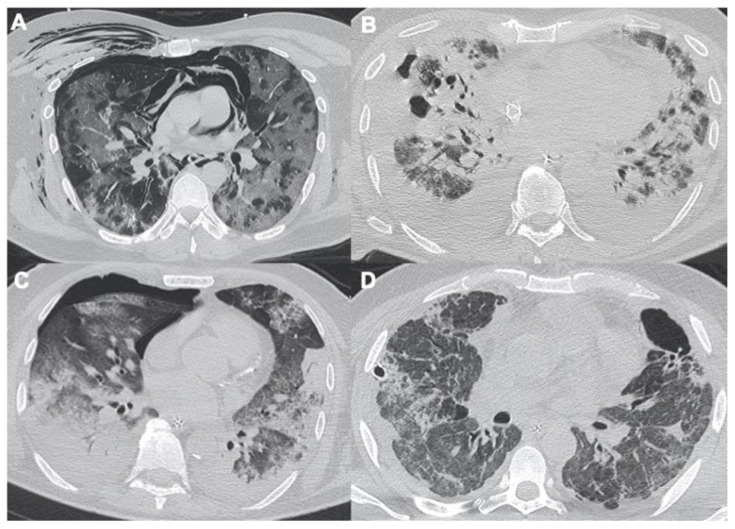
Three types (**A**–**C**) of barotrauma due to intensive care ventilation. (**A**) Pneumomediastinum associated with subcutaneous emphysema of the right pectoral region. (**B**) Middle lobe pneumatoceles. (**C**) Right pneumothorax. (**D**) Lingular bronchopleural fistula and chest tube on the right in late phase ARDS; architectural distortion caused by reticulation and traction bronchiectasis.

In the late phase of ARDS, CT images can show signs of fibrosis with traction bronchiectasis and reticulation caused by architectural distortion [33]. A typical complication is pneumomediastinum and pneumothorax caused by barotrauma, especially in intubated patients [34] (Figure 3). With a chest X-ray (for example, performed bedside during the follow-up), radiologists could quantify the ARDS extension using the Rale Score [14].

Its low cost, reproducibility, and the possibility of studying the heart and main vessels make lung ultrasound an important tool for monitoring COVID-19 patients; In particular, the use of ultrasound is useful in mechanically ventilated subjects to monitor the recruitment maneuvers, to evaluate the benefits of pronation–supination, and to detect complications such as pneumothorax or pleural effusion [35].

### 2.3. Pulmonary Embolism

**Figure 4:**
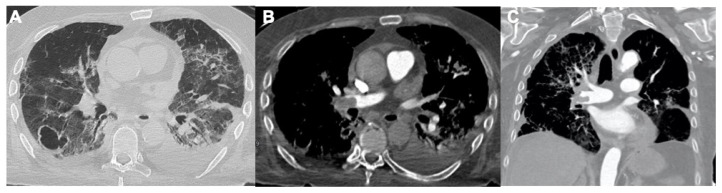
A 67-year-old male with acute pulmonary embolism related to COVID-19 infection. (**A**) Cavitation in right lower lobe associated with pericardial and pleural effusions. (**B**) Axial view of computed tomography pulmonary angiogram (CTPA) shows an acute filling defect in right main pulmonary artery. (**C**) Coronal views of same patient.

Another complication of SARS-CoV-2 infection is an increased risk of developing pulmonary embolism (PE). Several studies have shown a higher risk of developing thrombotic events, pointing out the need to evaluate D-Dimer and other coagulation tests during the management of these patients [36,37,38,39,40]. The incidence of PE in CT pulmonary angiography (CTPA) was reported to be 23–30% [41]. The emergency radiologist and pulmonary CT angiography are essential to identify the embolism, assessing the extent and its potential complications such as pulmonary infarction (Figure 4). With CTPA, there is the chance to have a PE score extension, which is extremely useful for clinicians, and it correlates optimally with the risk of exitus and future complications [42].

## 3. COVID-19 and Extrapulmonary Manifestations

### 3.1. Neurological Findings

**Figure 5:**
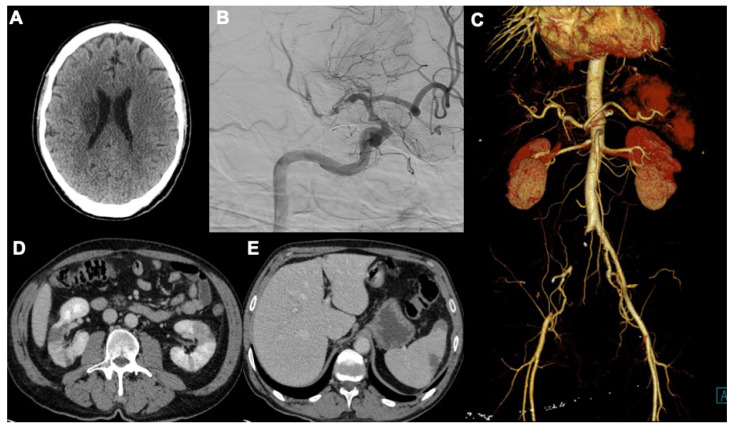
A case of acute thromboembolic arterial disease manifesting with acute right stroke (**A**), and angiography exam (**B**) showing focal occlusion of middle cerebral artery, due to complete occlusion of right common iliac artery in (**C**). Moreover, other complications occur: ischemic alterations of segmental areas of renal parenchyma (**D**) and splenic infarction (**E**).

**Figure 6:**
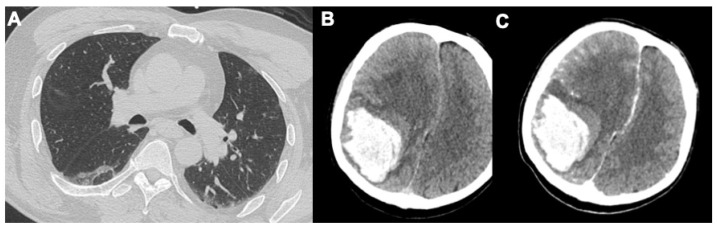
A case of spontaneous hemorrhage in a 56-year-old male. Lung involvement (**A**) is limited, but there is an extensive right parietal hemorrhage with an evident shift to the left, clear visible in baseline CT (**B**) and after contrast media injection (**C**).

Neurological symptoms caused by COVID-19 are most severe when they occur in the central nervous system (CNS), compared to the peripheral (PNS) one. The PNS symptoms are neuralgia, fatigue, myalgia, and Guillain–Barre syndrome [43]. Diagnostic imaging plays a marginal role in this kind of manifestation. CNS manifestations such as head pain, reduced consciousness, dizziness, acute cerebrovascular disease, ataxia, and epilepsy are recorded. The incidence of acute cerebrovascular complications was 5.7% in patients with severe SARS-CoV-2 infection; cerebrovascular disease is an independent predictor of mortality after COVID-19 [43,44,45] (Figure 5). Patients with SARS-CoV-2 infection who present CNS manifestation may undergo non-enhanced head CT to identify hemorrhage, venous sinus thrombosis, or stroke. About 33% of patients with acute or subacute COVID-19 may show brain abnormalities [46]. The results of a large French multi-institutional study showed that the most frequent CNS manifestations are ischemic strokes (27%), leptomeningeal potentiation (17%), and encephalitis (13%) [47,48]. An anticoagulant therapy can lead to the development of complications including traumatic or spontaneous intracranial hemorrhages (Figure 6). When a stroke is suspected, an MRI of the brain can be performed. In this setting, abbreviated MRI protocols can be adopted, limited to the acquisition of critical sequences such as diffusion-weighted imaging (DWI) and apparent diffusion coefficient (ADC) mapping, as well as axial T2-weighted fluid-attenuated inversion-recovery (FLAIR) sequences. When the suspect of viral encephalitis is high, contrast material can be helpful to assess leptomeningeal enhancement [49]. 

### 3.2. Cardiac Manifestations

**Figure 7:**
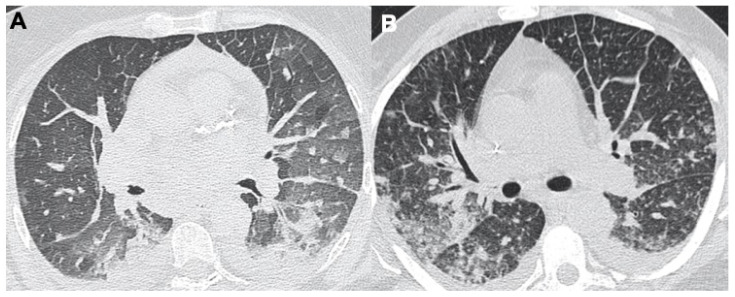
Two cases of congestive cardiac failure in COVID-19 patients. (**A**) Chest CT shows a bilateral interlobular septal thickening, cardiomegaly, pericardial effusion, bilateral pleural effusion, and broncho-vascular ground-glass opacities. (**B**) Bilateral pleural effusion, pulmonary interstitial oedema (interlobular septal thickening) and initial pulmonary alveolar oedema on the right thorax.

Angiotensin-converting enzyme 2 (ACE2), as a target receptor for SARS-CoV-2, is significantly expressed in the heart. Cardiovascular manifestations of COVID-19 include the elevation of cardiac biomarkers (ischemic or non-ischemic etiology), arrhythmia, arterial and venous thromboembolism (VTE), cardiogenic shock, and arrest [50]. Several cases of COVID-19 myocarditis have been observed, some of which were fatal. Like other viral agents, the pathophysiology of COVID-19 myocarditis is the result of both cell infection damage and auto-immune reaction [51]. Imaging plays a pivotal role in the early diagnosis of disease as well as detection of cardiovascular complications in SARS-CoV-2 infection. Although signs of cardiac failure can be readily evaluated via chest radiography, chest CT, and echocardiography, myocardial injury can be best assessed using cardiac MRI [49]. Chest X-ray and CT are useful in identifying pulmonary manifestations related to heart failure. Pleural effusions, vascular congestion, perihilar and interstitial oedema, and diffuse hazy opacities are typical in this kind of patient [52] (Figure 7).

### 3.3. Abdominal Manifestations

**Figure 8:**
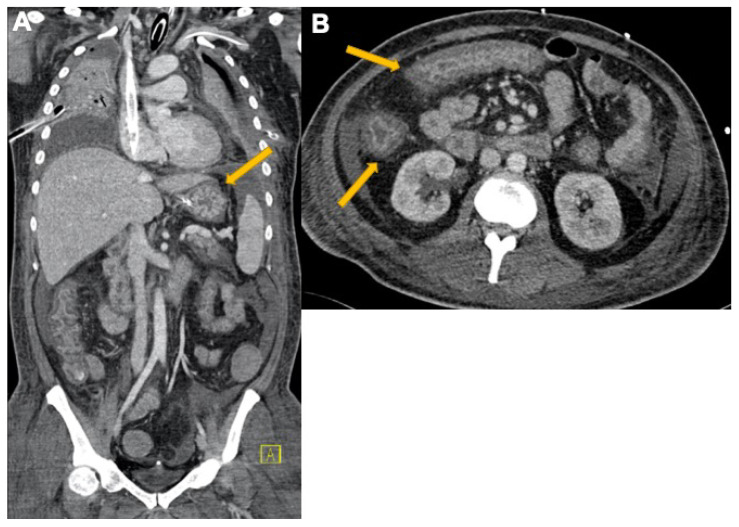
A 58-year-old male admitted to intensive care. (**A**) CECT coronal scan in a patient with gastritis (yellow arrow) and colitis. Free fluid in right and left subphrenic space, among paracolic gutters and pelvis. (**B**) Axial view shows (yellow arrows) COVID-19-related colitis. Large bowel lumen collapsed due to wall thickening, low-density submucosal oedema, and bilateral paracolic gutter ascites.

Among the most frequent gastrointestinal (GI) symptoms of SARS-CoV-2 infection, there are nausea, diarrhea, vomiting, and abdominal pain. At initial presentation, some COVID-19 patients had only GI symptoms without pulmonary manifestations [53]. Involvement of the GI and abdominal viscera appears to be related to the expression of ACE2 in the gastrointestinal tract and, although to a lesser extent, also in the biliary epithelium. Small and large bowel involvement is one of the most common GI manifestations; among possible explanations could be the high expression of ACE2 receptors on enterocytes membranes, which are considered the main virus cellular carriers [54] (Figure 8). 

**Figure 9:**
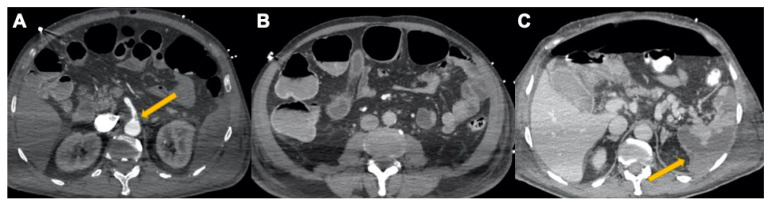
COVID-19-related abdominal vascular disease. (**A**) CECT in arterial phase, yellow arrow shows a partial thrombosis of the superior mesenteric artery. (**B**) CECT in venous phase, colic air-fluid level with middle colic distension and pericolic fat inflammation. (**C**) After 2 days, CECTs show pneumoperitoneum and multiple spleen infarcts (yellow arrow).

**Figure 10:**
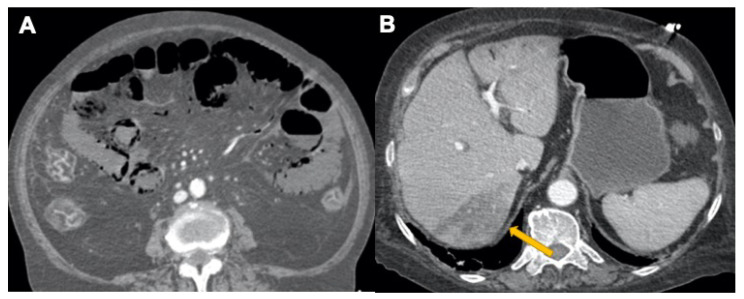
Abdominal vascular disease COVID-19 related in a 58-year-old female patient. CECT venous phase shows: (**A**) intramural bowel gas of small intestine associated to superior mesenteric vein branches gas and large bowel lumen collapsed due to wall thickening. (**B**) Yellow arrow shows hepatic ischemia of segment 7 associated with left lobe peripheral ischemia and portal branch gas.

**Figure 11:**
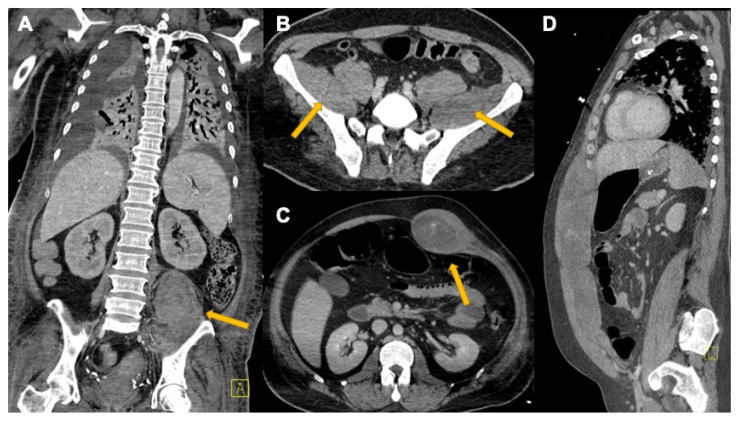
COVID-19-related spontaneous muscular hemorrhage in two patients: (**A**,**B**) show spontaneous bleeding in both iliac muscles (arrows); (**C**,**D**) show an important hemorrhage into the left abdominal wall. In both of these cases, there are no CCT signs of active bleeding.

Cytokine storm, endothelial activation, and inflammation caused by SARS-CoV-2, can provoke bowel ischemia, indicated by the presence of fibrin clots in major mesenteric vessels [55]. Contrast-enhanced CT (CECT) is the modality of choice for detecting intestinal involvement. The most typical CT findings include intestinal dilation, abdominal free fluid, fat stranding, intestinal wall thickening, a low-density ring of submucosal oedema between mucosa, and increasing serosa. Later findings are bowel wall pneumatosis and the absence of mucosal enhancement [56] (Figure 9 and Figure 10). Liver injury in SARS-CoV-2 infection could be related to a direct cytopathic effect of the virus in the liver. Elevated levels of liver enzymes such as AST, ALS, and GGT can be found in many patients’ blood (62.4%) [57]. While studying the abdomen, it is not rare to find vascular-related alterations such as muscular spontaneous hemorrhage, due to COVID-19-related coagulopathy (Figure 11).

### 3.4. Other Manifestations

Numerous studies have shown the relationship between acute kidney injury and SARS-CoV-2 infection, clinically and pathologically, by the development of microangiopathy, acute tubular necrosis, interstitial inflammation, and collapsing glomerulopathy [58,59,60]. Acute renal failure occurred frequently in critically ill patients admitted to intensive care, with an incidence ranging from 78% to 90%. Additionally, hematuria and proteinuria have been reported in nearly half of COVID-19 patients [61]. SARS-CoV-2 can cause also cutaneous manifestations including acral areas of erythema with vesicles or pustules (19%), maculopapular eruptions (47%), urticarial lesions (19%), vesicular eruptions (9%), and livedo reticularis and necrosis (6%) [62]. An interesting review of the ocular manifestation of COVID-19 shows that the prevalence is 11%. The most frequent ocular symptoms and diseases in COVID-19 infection are dry eyes or foreign body sensation (16.0%), redness (13.3%), tearing (12.8%), itching (12.6%), eye pain (9.6%), and discharge (8.8%) [63].

## 4. Conclusions

The COVID-19 pandemic has caused millions of deaths around the world. Pulmonary involvement is the most significant aspect of the disease. However, the heterogeneity of the clinical manifestations and multi-organ involvement makes the course of the disease extremely insidious. Understanding the pathogenetic mechanisms and the imaging characteristics is fundamental, especially in the emergency setting. Systemic vascular involvement due to COVID-19 has to be recognized and treated promptly to avoid deaths: ischemic and hemorrhagic lesions are frequent in every human organ, even if pulmonary involvement is not extensive. Through imaging, the early identification of multisystem involvement and related complications make the figure of the radiologist essential in the patient’s diagnostic and therapeutic algorithm.

## Data Availability

Not applicable.
